# Integrating aesthetics and efficiency: AI-driven diffusion models for visually pleasing interior design generation

**DOI:** 10.1038/s41598-024-53318-3

**Published:** 2024-02-12

**Authors:** Junming Chen, Zichun Shao, Xiaodong Zheng, Kai Zhang, Jun Yin

**Affiliations:** 1 Faculty of Humanities and Arts, Macau University of Science and Technology, Taipa, 999078 Macau; 2 School of Design, Jiangnan University, Wuxi, 214000 China

**Keywords:** Engineering, Computational science, Computer science, Information technology

## Abstract

The interior design suffers from inefficiency and a lack of aesthetic appeal. With the development of artificial intelligence diffusion models, using text descriptions to generate aesthetically pleasing designs has emerged as a new approach to address these issues. In this study, we propose a novel method based on the aesthetic diffusion model, which can quickly generate visually appealing interior design based on input text descriptions while allowing for the specification of decorative styles and spatial functions. The method proposed in this study creates creative designs and drawings by computer instead of from designers, thus improving the design efficiency and aesthetic appeal. We demonstrate the potential of this approach in the field of interior design through our research. The results indicate that: (1) The method efficiently provides designers with aesthetically pleasing interior design solutions; (2) By modifying the text descriptions, the method allows for the rapid regeneration of design solutions; (3) Designers can apply this highly flexible method to other design fields through fine-tuning. (4) The method optimizes the workflow of interior design.

## Introduction

Most people dream of owning an aesthetically pleasing home, and living in such a home can make the occupants feel cheerful^1^. Creating an aesthetically pleasing home usually requires the help of a professional designer, who must use their own aesthetic and professional skills to complete the design for the client. However, designers face two significant problems when designing interiors. On the one hand, designers must create interior designs with different decoration styles for customers to choose from. The huge workload and continuous design revisions lead to low efficiency^2–6^. On the other hand, it is a significant challenge for designers to create aesthetically pleasing designs in a limited time^3,7^. Therefore, the key is to solve the problem of low efficiency and a lack of aesthetic appeal in interior design.

The diffusion model has developed rapidly in recent years^8–12^. Due to its excellent image-generation ability, it has become the mainstream generation model^8–10^. The diffusion model completes model training by learning a large amount of pairing information between text descriptions and images^12–15^. Diffusion models can batch-generate high-quality and diverse images from input text descriptions^10,16–19^.

While diffusion models perform well in most domains, there is still room for improvement in the technically demanding field of interior design. Two areas need improvement. On the one hand, the traditional diffusion model does not consider aesthetic factors^1,20^, resulting in most of the interior designs generated by the diffusion model lacking aesthetic appeal. On the other hand, traditional diffusion models use big data collected from the internet for model training, but most of the data lack professional annotations^21–23^. For example, there is a lack of accurate annotation of interior decoration style and spatial function in big data, which leads to confusion in the interior design decoration style and spatial functions generated by diffusion models trained with these data. Therefore, it is necessary to improve the diffusion model in the design field, and it is essential to add aesthetics, decoration style, and spatial function control to the diffusion model.

**Figure 1:**
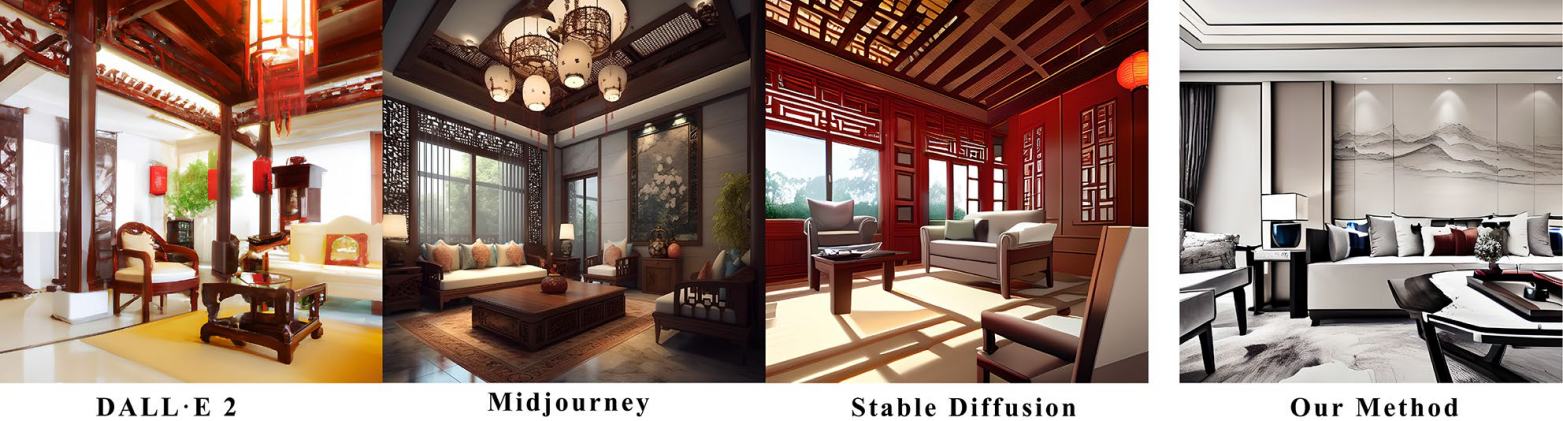
Comparison between mainstream diffusion models and our method for generative living-room design. The images generated by Dall$\cdot$E 2 (left) exhibit incorrect spatial sizes, furniture placement, and decoration styles. Midjourney (second from left) produces erroneous lighting fixtures and unrealistic images. Stable Diffusion (third from left) needs to solve the problem of decoration style and furniture generation. None of these images meet the requirements for interior design. In contrast, our proposed method (far right) addresses these issues. (Prompt word: “Realistic, Chinese-style living room with a touch of modernity, featuring a sofa and a table”).

This research enhances the traditional diffusion model by introducing a fresh and comprehensively annotated indoor dataset with aesthetic scores, decoration styles, and space functions. It further innovates by proposing a unique compound loss function, supplementing the model with aesthetics, decoration styles, and spatial functions while retraining it. This improvement enables the enhanced model to generate interior designs that are aesthetically pleasing and specify the decoration style and function of the space. The upgraded diffusion model can effectively address the prevalent issues of insufficient aesthetically pleasing designs and low productivity in interior design. Figure 1 shows a comparison between our technique for generating interior design effects and the present mainstream diffusion models, including Dall$\cdot$E 2^24^, Midjourney^25^, and Stable Diffusion^26^.

**Figure 2:**
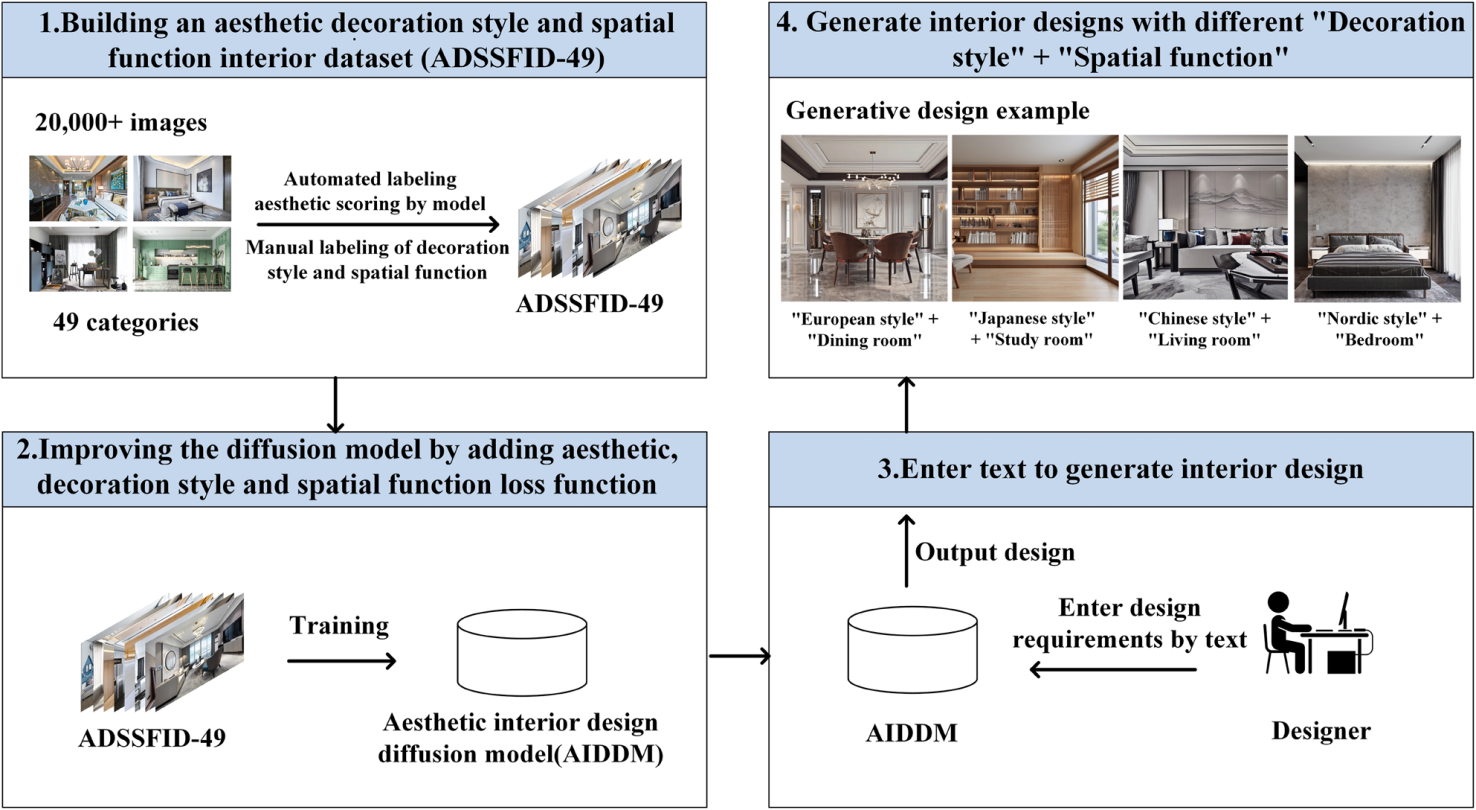
Research framework. We first collected over 20,000 indoor design images and annotated them with aesthetic scores, decoration styles, and spatial functionality to create the ADSSFID-49 dataset. We then fine-tuned a diffusion model to generate aesthetically pleasing interior designs. Designers can input design requirements, including decoration styles and functional needs, in the form of text into the model to obtain their desired designs.

Further explanation of our method. We first collected a dataset called the aesthetic decoration style and spatial function interior dataset (i.e., ADSSFID-49) to address the problem of a lack of training data. This dataset automatically annotates the aesthetic scores of each image using an aesthetic evaluation model and manually annotates the interior decoration style and spatial function of each image. Then, we proposed a compound loss function that comprehensively considers aesthetics, decoration style, and spatial function. This function enables the diffusion model to learn the decoration style and spatial function information of the interior design during the training process and ensures that the generated design is aesthetically pleasing. We trained the model using fine-tuning, requiring fewer data than retraining the entire model, significantly reducing training time and cost. The trained model is called the aesthetic interior design diffusion model (i.e., AIDDM). The AIDDM can automatically generate batches of interior designs with aesthetics, correct decoration styles, and spatial functions for designers to choose from. The AIDDM increases interior design efficiency, reduces the difficulty of achieving aesthetically pleasing designs, and revolutionizes the design workflow. The framework of this research is shown in Fig. 2.

**Figure 3:**
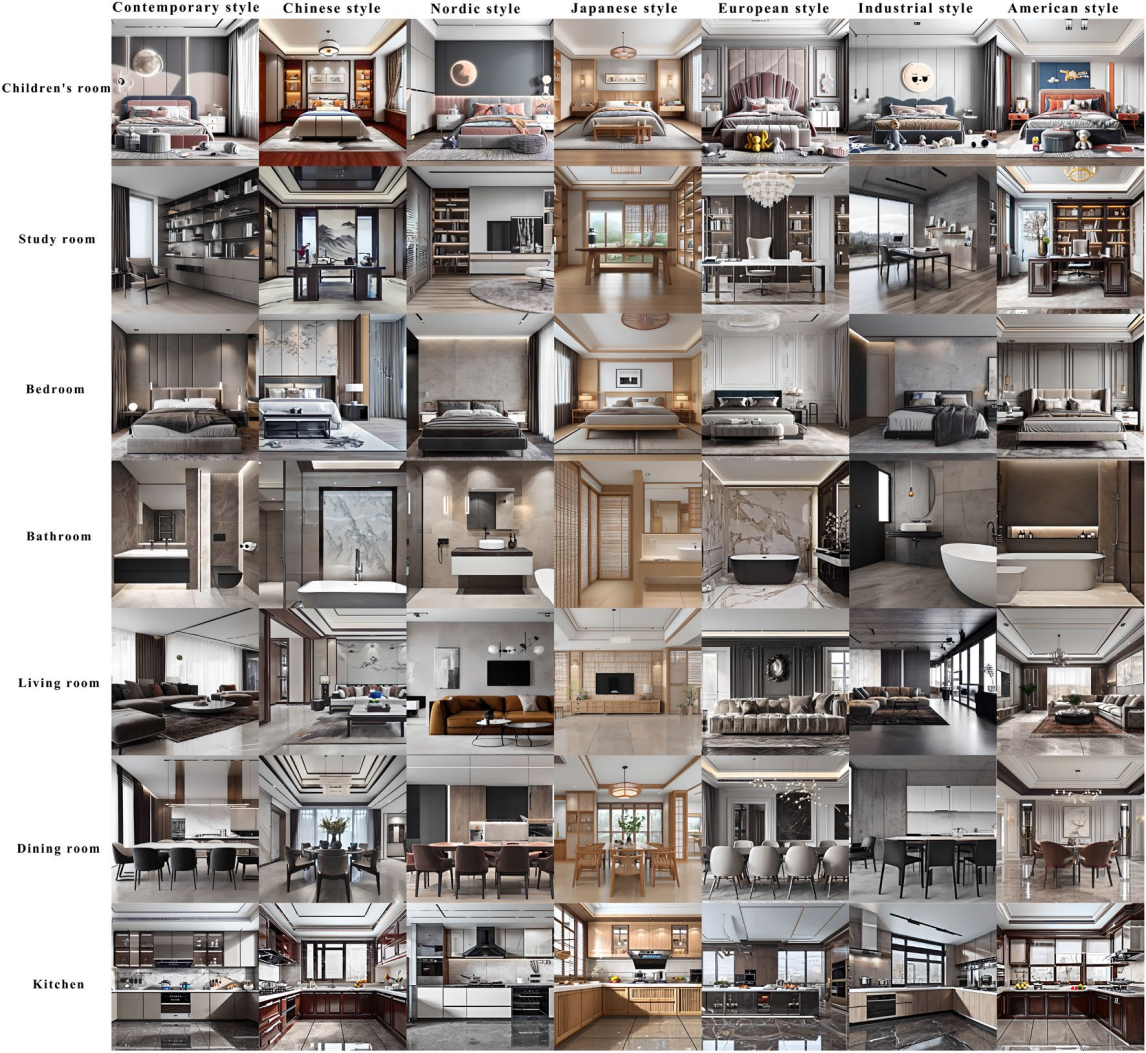
Our diffusion model generates designs for different decorative styles and spatial functions. The x-axis represents seven distinct decoration styles, while the y-axis represents seven different spatial functionalities. The combination of these variables allows for the creation of 49 common types of interior designs. Our method incorporates aesthetic considerations to ensure that the generated images have a certain level of aesthetic appeal.

The AIDDM model proposed in this research can generate aesthetically pleasing interior designs with specified decoration styles and spatial functions. Figure 3 demonstrates the effects of generating interior designs with different decoration styles and spatial functions.

The main contributions of this research are as follows:

1. Proposing an integrated diffusion model with aesthetic control can generate aesthetically pleasing designs.
2. Proposing an innovative workflow for generating interior designs based on text.
3. Proposing a new composite loss function that improves the generation effectiveness of the diffusion model.
4. Creating a new interior dataset with aesthetic scores, decoration styles, and spatial functions.
5. Demonstrating the advantages of our method in generating interior designs by comparing it with other popular diffusion models.

## Literature review

### Challenges of traditional interior design

Interior design refers to the creation of spaces within a building by designers^3,27^. It is a challenging task. Designers must consider regulations, spatial functionality planning, color schemes, and material selection to shape the decoration style^6,7^. In addition to ensuring that the spatial layout and decoration style are satisfactory to clients, designers need to ensure that the design is aesthetically pleasing. An aesthetically pleasing design brings emotional delight^3^.

A key challenge in interior design lies in the inefficiency of the process. The traditional interior design workflow is highly complex, involving multiple steps such as requirements analysis, client communication, conceptual design, spatial layout, material and furniture selection, and rendering^3,27^. Due to the complexity of interior design, even minor modifications often require the designer to repeat the entire design process. This linear workflow leads to repetitive design work, resulting in a decrease in design efficiency^27^. Furthermore, clients often request multiple design options for the same space in order to choose the most satisfactory design. This practice significantly increases the workload for designers, especially when they have to meet client demands within a limited time frame. As a result, designers often find themselves working overtime to meet deadlines.

Another significant challenge in interior design is the attainment of aesthetically pleasing designs^1,4^. Interior designers must factor in elements such as spatial layout, color harmony, material choice, furniture arrangement, and lighting design, among others. Designers require a blend of creativity, artistic sensibility, and practical understanding of the design. They need to perpetually research and master cutting-edge design trends and technologies to sustain their innovation and competitiveness, which poses further challenges^3,6,7^.

Hence, enabling designers to efficiently produce aesthetically pleasing interior designs in bulk is crucial in tackling the aforementioned challenges.

### Text-to-image diffusion model

Diffusion models for image generation have recently gained substantial attention^8,28–30^. Researchers continue to improve the performance of diffusion models, making them new mainstream generative models and demonstrating excellent image-generation capabilities^30–32^. A diffusion model mainly consists of a diffusion module and a denoising module. The diffusion module converts the input raw image into a noisy image by continuously adding noise. This is followed by a denoising module, which restores the noisy image to the original image. Through learning from ample data, the denoising module enables the diffusion model to remove noise from images, thus generating images^8,13,30^. Additionally, diffusion models offer control over the image-generation process. For generating images with specific features, text guidance can be incorporated during the denoising process. Converted into a computer-readable language, this guidance influences the entire image-generation process, ensuring alignment between the generated images and the text guidance, achieving controlled image-generation^32–36^. The simplicity of operation, through controlling image generation with textual descriptions alone, is a notable advantage of text-guided diffusion models.

However, conventional diffusion models face challenges, particularly performing poorly in certain professional domains due to the requirement for domain-specific paired image-text data for training. Enhancing diffusion models to improve their performance and usability is a necessary solution^37,38^.

There are two main methods for improving diffusion models. The first method is to retrain the entire diffusion model, which requires substantial training data and computational resources^24–26^. The second method is fine-tuning the diffusion model to enhance its performance in specific domains^37–40^. Considering the potential difficulty of obtaining high-quality training data in the field of interior design, fine-tuning the diffusion model is a more feasible choice.

There are four common methods for fine-tuning the model. The first, Textual Inversion^24,32,37^, does not change the weight of the original diffusion model but embeds new knowledge into the original model by providing the most suitable embedding vector for the new training data. This model can be trained quickly but produces mediocre results. The second method, Hypernetwork^38^, affects the image-generation results by adding additional networks in the middle layers of the original diffusion model. The third method is LoRA^39^, which changes the image-generation effect by exerting influence on the cross-attention layer of the diffusion model, yielding better results than Textual Inversion and Hypernetwork. Its model size is approximately 200 MB. The fourth method, Dreambooth^40^, adjusts the weights of all neural network layers. This method stipulates specific description words for the training images, avoiding language drift issues by ensuring these words are not conflated with other images and prompts during training^41,42^. It then designs a new loss function, the prior-preservation loss, to prevent overfitting during training. Models trained using this method are able to generate subject-specific images while preserving the fundamentals of the original model. Using this method requires only a small number of images and corresponding text descriptions to complete training, thereby improving the quality of image generation of the model in specific domains. Among these four methods, fine-tuning the model typically yields the best generation results.

### Public dataset

Open datasets serve as critical catalysts in the swift evolution of artificial intelligence, with numerous studies leveraging these publicly accessible resources. One example is the ImageNet image dataset^43^, which comprises over 20,000 categories and more than 10 million images. This vast dataset forms a valuable basis for tasks such as classification and segmentation. Referenced by over 60,000 researchers, it propels advancements in the field of artificial intelligence. Another case in point is the Berkeley Deep Drive-X (i.e., BDD-X) dataset^44^, which is currently the largest and most diverse driving video dataset. This dataset underpins many autonomous driving competitions, stimulating the development of autonomous driving technology. The Common Objects in Context (i.e., COCO) dataset^45^ is yet another significant dataset, consisting of images furnished with semantic and image annotation information. This dataset significantly contributes to computer vision advancements and has emerged as a benchmark for assessing image semantic understanding.

Currently, there is a lack of indoor datasets annotated with aesthetic scores and decoration styles. This deficit has hampered the progress of text-to-image generation models in interior design, resulting in models incapable of generating designs that are aesthetically pleasing aligned with defined decoration styles and spatial functionality. Hence, developing a new dataset incorporating aesthetic evaluations, interior design styles, and spatial functionality is imperative.

## Methodology

In recent years, significant breakthroughs have been made in the field of text-to-image generation using diffusion models. Models such as Dall$\cdot$E 2^24^, Midjourney^25^, Stable Diffusion^26^, and Dreambooth^40^ have emerged as prominent image-generation models in the past few years. These models have demonstrated remarkable performance in various application scenarios. However, there is still potential for improvement in the performance of diffusion models, particularly in generating aesthetically pleasing interior designs with specified decoration styles. This is especially relevant in the field of interior design.

In this research, we propose an improved aesthetic diffusion model for generating batches of aesthetically pleasing interior designs. Our method comprises a self-created dataset called the aesthetic decoration style and spatial function interior dataset (i.e., ADSSFID-49), which includes information on aesthetic scores, decoration styles, and spatial functionality in interior design. Additionally, we introduce a novel composite loss function that combines aesthetic scores, decoration styles, and spatial functionality as loss terms. By fine-tuning the model using this dataset and loss function, we have achieved the capability to generate interior design models that are aesthetically pleasing and aligned with specified decoration styles and spatial functionality in bulk. This method enhances the practicality of diffusion models in the field of interior design, as designers can obtain corresponding design results by simply inputting their design requirements in text form. This method offers a fresh design method for interior designers.

The proposed method follows a four-stage process. The first stage involves establishing the dataset. In the second stage, a new loss function is designed. The third stage focuses on fine-tuning the model using the dataset and the new loss function. Finally, in the fourth stage, designers utilize the model to generate and modify designs according to their requirements.

In the data collection stage, to address the need for interior design datasets with aesthetic scores, this research involved professional designers gathering over 20,000 high-quality interior design images from renowned interior design websites. Next, we employed state-of-the-art aesthetic score models to automatically rate these images and mapped the score distribution to integers ranging from 1 to 10. Subsequently, professional designers annotated each image with decoration styles and spatial functionality. Through this process, we successfully established an interior dataset, named ADSSFID-49, which includes aesthetic scores, decoration styles, and spatial functionality annotations.

During the loss function construction phase, this study introduces a novel composite loss function. This function incorporates aesthetic scores, decorative styles, and spatial functions as additional losses (Eq. 2), building upon the foundation of the diffusion model^′^s conventional loss function (Eq. 1): model training is primarily aimed at producing interior designs that exhibit predetermined aesthetic scores, decorative styles, and spatial functionalities. The model undergoes training to minimize the loss, thereby attaining the specified capabilities.

The basic diffusion model is given by Eq. (1):

1 $$\begin{aligned} {\mathbb {L}}_{\varvec{{Y}},\varvec{{h}},\varvec{{\epsilon }},\varvec{{t}}}[w_t||{\hat{Y}}_\theta (\alpha _tY+\sigma _t\epsilon ,\varvec{{h}})-Y||_2^2] \end{aligned}$$

In Eq. (1), ${\mathbb {L}}$ represents the average loss, and model training aims to decrease this value. A lower loss indicates better image-generation quality. ${\hat{Y}}_\theta$ refers to the evolving diffusion model that continuously receives a noisy image vector $\alpha _tY+\sigma _t\epsilon$ and a text $\varvec{{h}}$, and produces a predicted image. This predicted image is compared to the truth image *Y*, and the difference between them is quantified as the loss. The error between the predicted and ground truth images is measured using the squared loss. $w_t$ is the weight parameter used to control the weight change of the diffusion model in different time periods. The external *N* represents the accumulation of losses from all the images, which is then divided by the total number of images to obtain the average loss per image. During training, the diffusion model adjusts its parameters to reduce the discrepancy between the generated and truth images, ultimately minimizing ${\mathbb {L}}$.

The composite loss function proposed in this research is given by Eq. (2):

2 $$\begin{aligned} {\mathbb {L}}_{\varvec{{Y}},\varvec{{h}},\varvec{{\epsilon }},\varvec{{\epsilon ^{'}}},\varvec{{t}}}[w_t||{\hat{Y}}_\theta (\alpha _tY+\sigma _t\epsilon ,\varvec{{h}})-Y||_2^2+\lambda {w_{t^{'}}}||{\hat{Y}}_\theta (\alpha _{t^{'}}Y_{pr}+\sigma _{t^{'}}\epsilon ^{'},\varvec{{h_{pr}}})-Y_{pr}||_2^2] \end{aligned}$$

The improved loss function (Eq. 2) addresses the limitations of the traditional diffusion model in generating aesthetically pleasing designs with different decoration styles. Equation (2) combines aesthetic score, decoration style, spatial functionality, and prior knowledge as components of the loss function, building upon Eq. (1). Equation (2) consists of two main components. The first component measures the discrepancy between the images generated by the trained model and the ground truth images. ${\hat{Y}}_\theta$ represents the new diffusion model, which incorporates aesthetic score, decoration style, and spatial functionality losses. The difference between the images generated by this model and the ground truth images *Y* contributes to the loss of the first component. The second component is the prior knowledge loss, which compares the images generated by the new diffusion model (i.e., ${\hat{Y}}_\theta (\alpha _{t^{'}}Y_{pr}+\sigma _{t^{'}}\epsilon ^{'},\varvec{{h_{pr}}}$)) with those generated by the pre-trained diffusion model (i.e., $Y_{pr}$). A smaller difference between these images indicates that the newly trained model retains the general knowledge of the base model. $\lambda {w_{t^{'}}}$ is a weight that can be automatically learned to adjust the contributions of these two components, aiming to achieve better generation results. The combination of the first and second component losses allows the new diffusion model to retain the general knowledge of the pre-trained model while learning aesthetic, decoration style, and spatial functionality knowledge. As a result, the fine-tuned diffusion model can generate aesthetically pleasing interior designs with specified decoration styles and spatial functionality.

Using Stable Diffusion V1.5 as the foundational model in the fine-tuning phase of the diffusion model and as a baseline for subsequent qualitative and quantitative comparisons. We utilized the ADSSFID-49 dataset to fine-tune the improved diffusion model. Specifically, the improved diffusion model employed a new composite loss function to learn from this dataset, continuously reducing the loss during training. This allowed the model to acquire knowledge in respect of the aesthetic score, decoration style, and spatial functionality, resulting in a new diffusion model for aesthetic interior design, known as the aesthetic interior design diffusion model (i.e., AIDDM).

**Figure 4:**
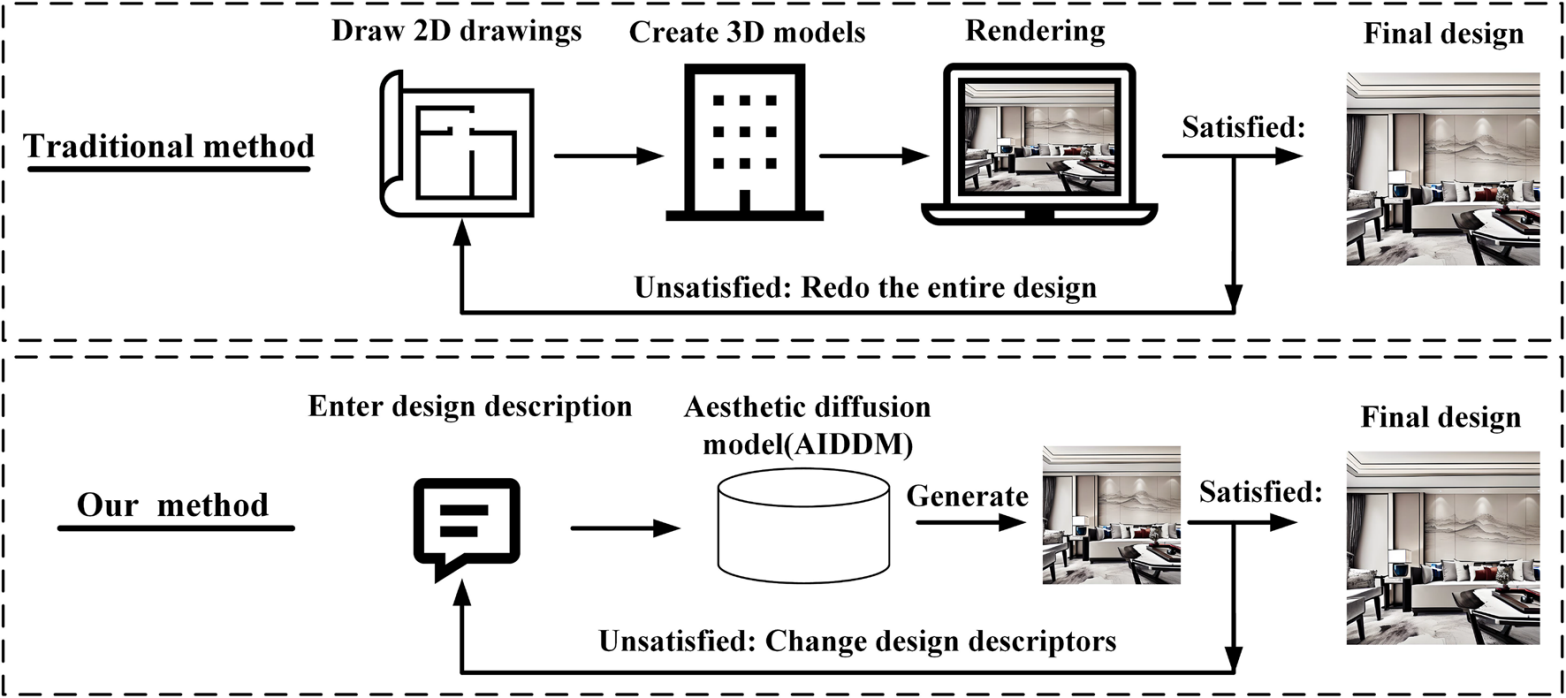
Comparison of the design process between different design methods. Conventional methods in the design stage require drawing 2D plans, creating 3D models, applying materials to the models, and rendering visualizations. In contrast, our method only requires textual descriptions to generate design visualizations directly. In the modification stage, conventional methods require repeating the entire design process, while our method only requires modifying the textual prompts to regenerate the design.

During the model utilization stage, designers can use the AIDDM for design generation and modification. In the design generation phase, users only need input textual descriptions of their desired decoration style and spatial functionality to generate an interior design. This method allows for the rapid and batch generation of interior designs with different decoration styles for users. Compared to traditional methods, our proposed method eliminates cumbersome workflow steps such as drawing 2D plans, creating 3D models, texturing, and rendering, thereby significantly improving design efficiency. Traditional design processes often take several days to complete a single design. In contrast, our method can generate a design in approximately two seconds on a computer with 24 GB of graphics memory, resulting in around 30 designs per minute. In the design modification stage, our method only requires changing the design prompts to regenerate a design without repeating the entire design process. Therefore, it offers advantages in optimizing the design workflow and enhancing design efficiency. Our method reduces the difficulty of creative design by generating designs in bulk, thus accelerating the design decision-making process. Figure 4 illustrates the differences between traditional design methods and our proposed method.

## Experiments and results

### Implementation details

The diffusion model was trained on a computer with a Windows 10 operating system. The computer had 64GB of RAM and used an NVIDIA 3090 graphics card with 24 GB of memory. The training software used was PyTorch, with each image undergoing 100 iterations. The image preprocessing method involved the computer resizing the input images proportionally to a maximum resolution of 512 pixels on the longer side. Data augmentation was performed using horizontal flipping. The model’s learning rate was set to 0.000001, and the batch size was set to 24. Xformers and FP16 were utilized for accelerated computations. The total training time for fine-tuning the diffusion model was 20 hours.

### ADSSFID-49 dataset

This research aimed to generate a large quantity of aesthetically pleasing and specified decoration-style interior designs using text. Due to the lack of interior datasets with aesthetic scores, this study created the aesthetic decoration style and spatial function interior dataset (ADSSFID-49). Expert interior designers curated this dataset from reputable websites such as “3d66^46^,” “om^47^,” and “znzmo^48^.” Initially, the designers procured over 40,000 free, high-quality images from these sources. Subsequently, they meticulously evaluated each image, excluding those displaying incongruent decoration styles or unclear details. A stringent selection process was followed, and more than 20,000 images aligned with the established criteria. Furthermore, designers manually annotated the decoration styles and spatial functionalities depicted in these images. Ultimately, employing an open-source aesthetic evaluation mode^49^, aesthetic scores were assigned to each image, culminating in the formation of ADSSFID-49.

**Figure 5:**
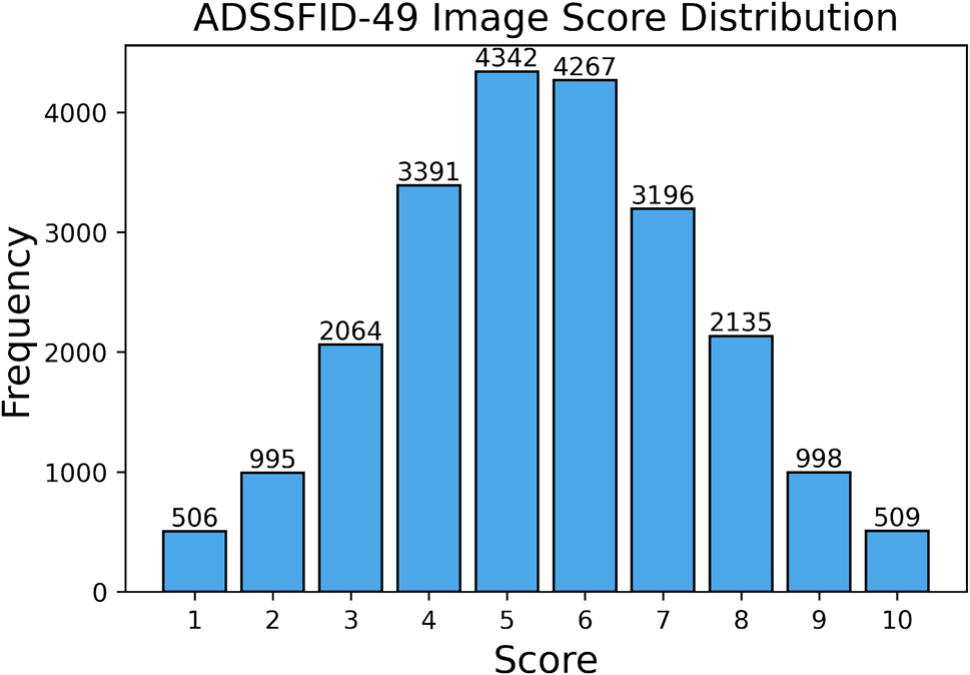
Distribution of aesthetic scores on the ADSSFID-49 dataset. The dataset uses an aesthetic scoring model to label the aesthetic score of each image automatically and maps all the scores to integers between 1 and 10, conforming to a normal distribution through a normalization method.

We employed a state-of-the-art aesthetic scoring model^49^ for the automated aesthetic annotation of interior design images. This model, proposed in 2023, was trained on a dataset of 137,000 images with aesthetic scores. The authors of this method indicate that their proposed model outperforms other mainstream models in terms of aesthetic score prediction^49^. We utilized this model to automatically annotate the aesthetic scores of each image in the ADSSFID-49 dataset. To make the diffusion model easier to train, we normalized all scores using a mapping that adheres to a normal distribution, resulting in integer scores between 1 and 10. The distribution of aesthetic scores for the processed ADSSFID-49 images can be seen in Fig. 5.

We enlisted the expertise of professional designers to manually annotate the decoration styles and spatial functions of the ADSSFID-49 dataset. The decoration style annotations encompass seven categories: “Contemporary style”, “Chinese style”, “Nordic style”, “Japanese style”, “European style”, “Industrial style”, and “American style”. The spatial function annotations also consist of seven categories: “Children’s room”, “Study room”, “Bedroom”, “Bathroom”, “Living room”, “Dining room”, and “Kitchen”. The distribution of the different categories of images is shown in Table 1.

**Table 1:** Image distribution of each decoration style and spatial function corresponding to the ADSSFID-49 dataset.

	Contemporary style	Chinese style	Nordic style	Japanese style	European style	Industrial style	American style	Total
Children’s room	422	393	391	295	227	130	250	2108
Study room	583	395	243	310	205	299	308	2343
Bedroom	852	693	726	314	614	489	390	4078
Bathroom	649	192	851	310	420	242	249	2913
Living room	956	865	1348	325	884	105	678	5161
Dining room	1399	467	737	381	675	231	420	4310
Kitchen	292	412	168	173	190	74	181	1490

**Figure 6:**
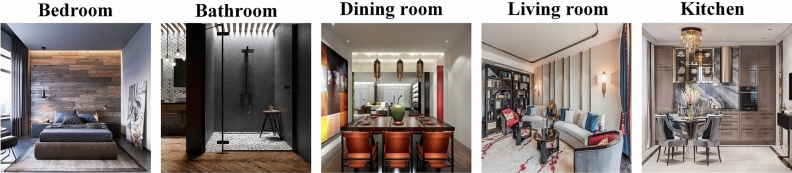
ADSSFID-49 training data sample display.

From Table 1, we can observe that in the ADSSFID-49 dataset, when sorted by a decoration style, the “Contemporary style” has the highest number of images (5153 images), while the “Japanese style” has the fewest (2108 images). When sorted by spatial function, the “Living room” category has the highest number of images (5161 images), while the “Kitchen” category has the fewest (1490 images). In total, there are 22,403 images in the dataset. Figure 6 shows some training data samples.

### Evaluation metrics

The evaluation of interior design involves subjective and objective assessments. Typically, conventional objective evaluation methods employ computerized techniques to assess image clarity and compositional coherence. However, considering that our focus in interior design evaluation is not solely on image clarity or compositional coherence but on the aesthetic appeal of the generated interior designs, the consistency of decoration styles, and the rationality of spatial functions, these aspects require subjective evaluations by professional designers. Therefore, we did not employ conventional objective evaluation methods^50,51^.

Assessing generative architectural design images poses a significant challenge. Traditional automated image evaluation methods fail to evaluate the design content effectively^50,51^ . Consequently, this study invited experienced industry designers to collaboratively discuss and formulate a series of evaluation metrics tailored for professional interior design. These metrics encompass eight categories: “aesthetically pleasing,” “decoration style,” “spatial function,” “design details,” “object integrity,” “object placement,” “realistic,” and “usability.”

Subsequently, the content and significance of the evaluation metrics designed herein are elucidated. We identify “aesthetically pleasing” and “usability” as the pivotal indicators. Specifically, “aesthetically pleasing” signifies that the generated design possesses aesthetic appeal, a crucial interior design aspect. The “usability” metric indicates that upon a comprehensive observation of the generated image, no apparent errors are observed, thus validating the image’s usability. For the other indicators, “decoration style” refers to the consistency between the generated interior design’s decorative style and the provided cues. “Spatial function” pertains to the appropriateness of the generated space size and its alignment with the described spatial functions. “Design details” denote the richness and complexity of design elements in the generated image. “Object integrity” ensures the absence of defects in the generated objects. “Object placement” evaluates the rationality of the generated furniture positioning. Finally, “realistic” indicates that the generated image closely resembles a photograph taken by a camera. These evaluation metrics enable a comprehensive assessment of the design quality and show the practical value of the generated interior design.

### Visual assessment

**Figure 7:**
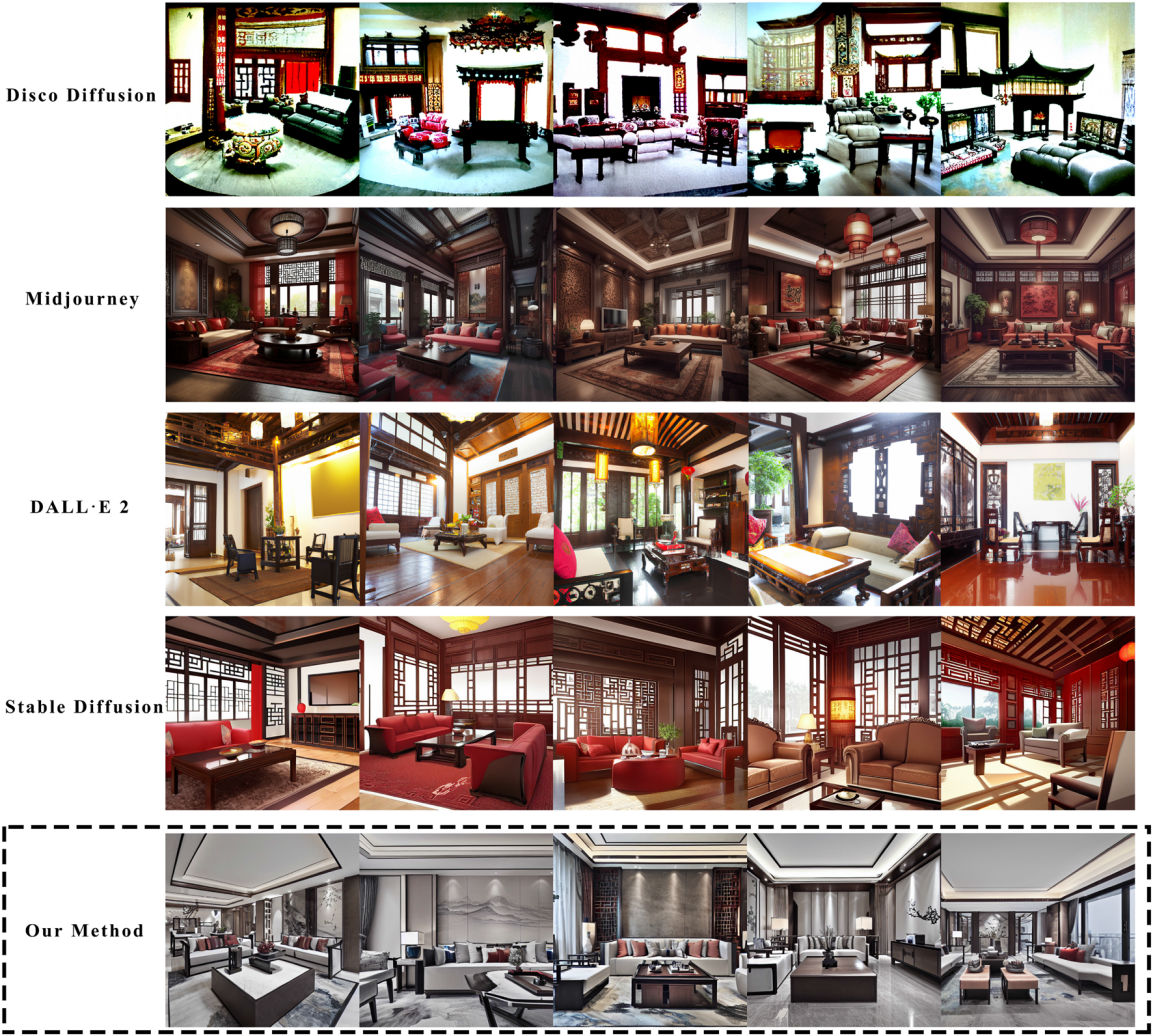
Other mainstream generative image methods compared with our method for generative design. (Prompt words: “Chinese-style living room, with a sense of modern design, a table and sofa, large angle, realistic, photo, high-definition”).

In this research, we visually compared our diffusion model with other popular diffusion models. We selected several mainstream diffusion models for comparison, including Disco Diffusion^52^, Dall$\cdot$E 2^24^, Midjourney^25^, and Stable Diffusion^26^. These are the most widely used and influential diffusion models, with active user counts exceeding one million^24–26^. We generated images of five Chinese-style living room designs using these models and performed a visual comparison. The generated images are shown in Fig. 7. By comparing these images, we can evaluate the differences in the effectiveness of different models in generating Chinese-style living rooms. This comparison will help us understand the strengths and areas for improvement of our diffusion model in generating interior designs.

By observing Fig. 7, we have drawn several conclusions regarding the performance of different methods. For Disco Diffusion^52^, this method failed to generate interior designs. It needed help comprehending the relationships between design elements and their connection to the space. The generated images needed more design details and aesthetic appeal. Midjourney^25^ demonstrated a better understanding of the relationships between design elements, resulting in images with some aesthetic appeal. However, Midjourney exhibited a bias in understanding decoration styles, leaning more towards ancient rather than modern styles. Additionally, the overall realistic of the images needed to be increased. Dall$\cdot$E 2^24^ produced highly realistic images. However, it needed an understanding of spatial function, object integrity, and object placement. These shortcomings affected the overall quality of the generated images. Stable Diffusion^26^ generated images with accurate spatial function and object integrity. However, it struggled with understanding decoration styles, leading to incorrect positioning of elements and a lack of aesthetic appeal. In summary, none of these methods fully satisfied the requirements for interior design in terms of aesthetic appeal, decoration style, spatial function, design details, object integrity, object placement, realistic, and usability. There is still room for improvement in applying these models in interior design.

Compared to other methods, the diffusion model trained in this research can simultaneously meet common design requirements. Table 2 presents the advantages and disadvantages of all the methods compared. Table 2 shows that the proposed method outperforms all the tested methods, with Midjourney^25^ ranking second, Stable Diffusion^26^ ranking third, and Dall$\cdot$E 2^24^ ranking fourth. Disco Diffusion^52^ is unsuitable for generating interior designs.

**Table 2:** Comparison of image-generation effects of different diffusion models.

Model	Aesthetically Pleasing	Decoration style	Spatial function	Design details	Object integrity	Object placement	Realistic	Usability
Disco Diffusion							$\checkmark$	
Midjourney	$\checkmark$		$\checkmark$	$\checkmark$	$\checkmark$	$\checkmark$		$\checkmark$
Dall$\cdot$E 2		$\checkmark$		$\checkmark$			$\checkmark$	
Stable Diffusion			$\checkmark$		$\checkmark$			$\checkmark$
Our Method	$\checkmark$	$\checkmark$	$\checkmark$	$\checkmark$	$\checkmark$	$\checkmark$	$\checkmark$	$\checkmark$

### Quantitative evaluation

**Figure 8:**
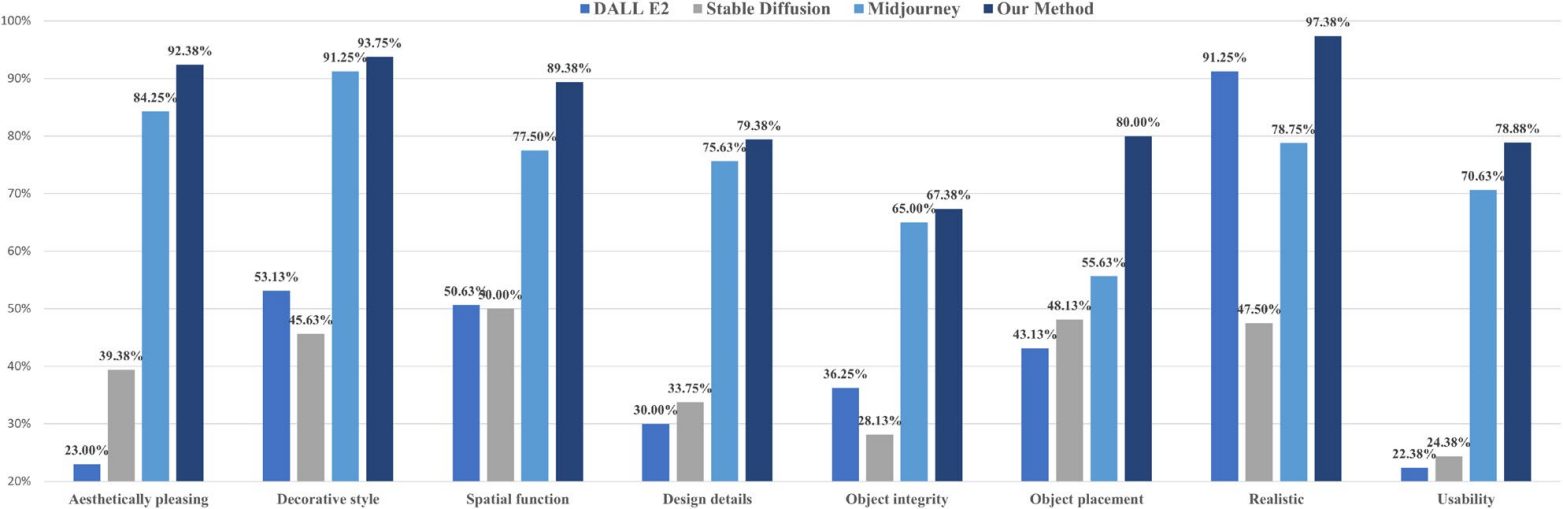
Different diffusion models generate quantitative assessments of interior design.

We generated 1,960 interior design images using Dall$\cdot$E 2^24^, Stable Diffusion^26^, Midjourney^25^, and the method proposed in this research (i.e., AIDDM). These images spanned 49 different categories, including seven decoration styles and seven spatial functionalities. Each category consisted of 10 generated images. To evaluate the quality of these images, we enlisted seven professional designers. The evaluation criteria included “aesthetically pleasing”, “decoration style”, “spatial function”, “design details”, “object integrity”, “object placement”, “realistic”, and “usability”. The evaluation process involved the experts judging whether the generated images met each criterion, awarding one point for compliance and zero points otherwise. Finally, we calculated the average score for each criterion by dividing the total score by the total number of images and converting it into a percentage. This allowed us to obtain quantitative scores for each model. The scores for different diffusion models are illustrated in Fig. 8:

From Fig. 8, it is evident that there are significant differences among the four models in generating interior designs. Our method outperforms Midjourney^25^, Dall$\cdot$E 2^24^, and Stable Diffusion^26^ in all the evaluation criteria. Compared to the model ranking second, our model shows significant advantages in the “Aesthetically pleasing”, “Spatial function”, “Object placement”, “Realistic”, and “Usability” criteria, exceeding them by 8.13%, 11.88%, 31.37%, 6.13%, and 8.25%, respectively. In particular, our model achieves high scores in the “Aesthetically pleasing”, “Decoration style”, and “Spatial function” criteria, demonstrating its ability to generate interior designs that are aesthetically pleasing and align with specified decoration styles and spatial functionalities.

We consider our method and the Midjourney model to be usable in generating interior designs. Midjourney^25^ achieved a usability score of 70.63%, while our model achieved 78.88%. Our method outperforms Midjourney^25^ regarding aesthetic appeal, appropriate spatial function, reasonable object placement, realism, and usability. However, Dall$\cdot$E 2^24^ and Stable Diffusion^26^ are considered unusable for interior design generation, with usability scores of only 22.38% and 24.38%, respectively.

### Generating design details showcase

**Figure 9:**
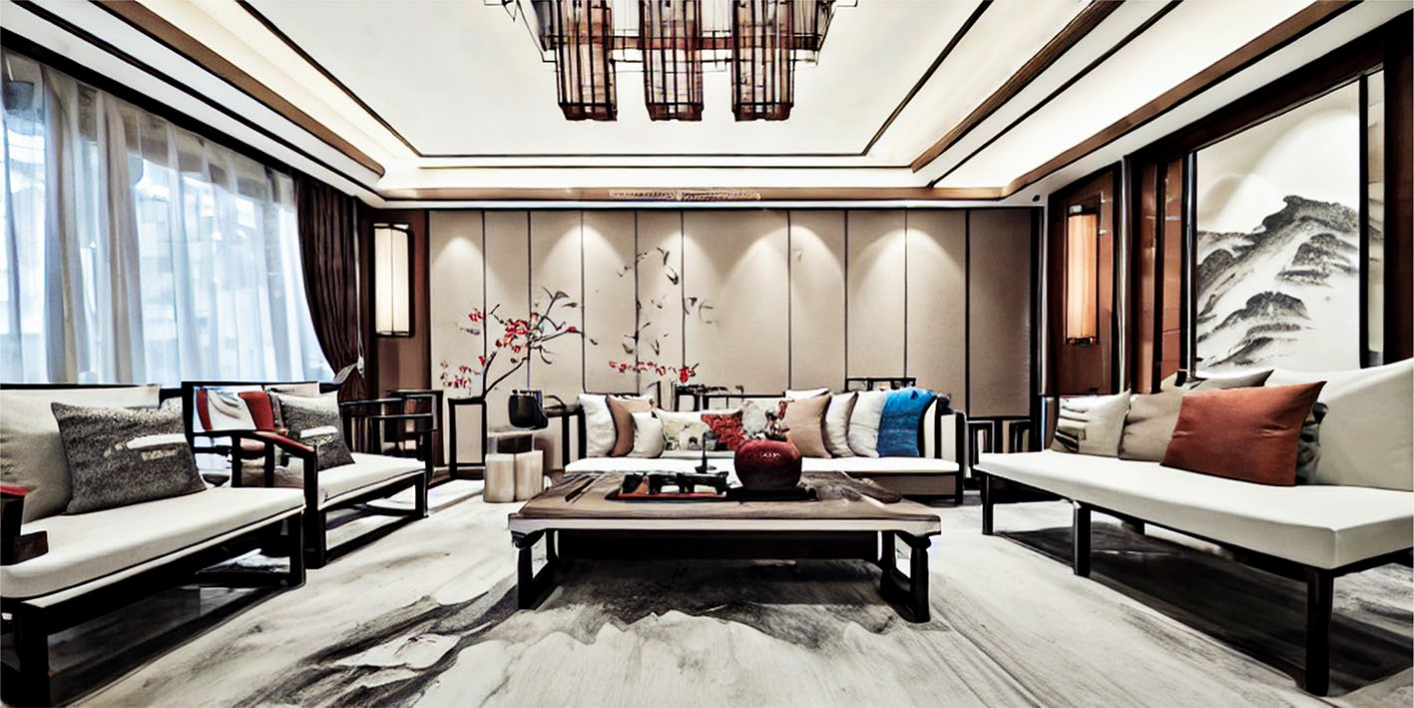
Detailed display of a Chinese-style study room generated by our trained diffusion model.

Figure 9 showcases a Chinese-style living room generated by our diffusion model. From the image, it is evident that the entire space possesses aesthetic appeal, and the decoration style and spatial function meet the requirements. This shows that our model is capable of generating designs with aesthetic appeal, specified decoration styles, and spatial function. Upon careful examination of the generated design details, we can observe that the furniture is placed in appropriate positions, with suitable dimensions, and the objects have no noticeable flaws. The image also includes numerous decoration items consistent with the design style, such as landscape paintings on the wall, tea sets, and vases on the coffee table, highlighting the model’s capability to generate detailed designs.

The images generated by the model exhibit a sense of realism, with well-handled lighting and shadow relationships. The light shining through the curtains into the room creates a soft and warm ambiance, while the recessed lights leave clear projections on the wall. However, there is still room for improvement in the model. For instance, the generation of lighting fixtures may be partially accurate, resulting in minor excess lines. Additionally, the projections of wall-mounted lights are irregular, as some areas exhibit lighting and shadow relationships without arranged lights. Despite these areas for improvement, overall, the interior designs generated using our diffusion model are usable and can enhance the efficiency of designers in generating design proposals and making design decisions.

## Discussion

This research provides comprehensive qualitative and quantitative evidence of the effectiveness of our method. In the qualitative research section, we visually compared our fine-tuned diffusion model with other methods, demonstrating its end-to-end capability in rapidly generating high-fidelity interior designs with diverse and specified decoration styles, surpassing the level achieved by other mainstream diffusion models. In the quantitative research section, we quantified the data to confirm that our method outperforms other methods in several metrics, particularly in the “Aesthetically pleasing”, “Spatial function”, “Object placement”, “Realistic”, and “Usability” categories, where we obtained a significant advantage, surpassing the second-ranked method by 8.13%, 11.88%, 31.37%, 6.13%, and 8.25%, respectively. Moreover, the high scores obtained in the “Aesthetically pleasing”, “Decoration style”, and “Spatial function” metrics further validate our model’s ability to generate aesthetically pleasing interior designs that align with specified decoration styles and spatial functions.

This research has introduced the aesthetic diffusion model, transforming the traditional linear workflow of interior design into a faster design acquisition method while avoiding cumbersome conventional design processes such as manual drawing, modeling, material mapping, and rendering. Our method demonstrates absolute advantages in design generation and modification efficiency. On a computer equipped with 24 GB of VRAM, our diffusion model can generate about 30 designs per minute, and modifying a design only requires the designer to change the prompt word to regenerate the design. In contrast, traditional design methods would take a week to complete an interior design, and modifying the design would consume another week. This clearly showcases the significant efficiency advantage of our method in design processes. Furthermore, our method can generate many aesthetically pleasing design options in different decoration styles for designers. This reduces the difficulty of creative design and accelerates the design decision-making process. In summary, our method is an innovative approach to interior design.

The proposed approach exhibits versatility: we have introduced a universally applicable fine-tuning model methodology and validated its efficacy through experimentation. Using this approach, researchers can collect datasets and transform diverse training objectives into loss functions, thereby facilitating the training of personalized diffusion models. As computational capabilities advance, individuals can feasibly train specific knowledge into personalized diffusion models. This methodology transcends the confines of interior design; for application in novel domains, one needs only to substitute the dataset and redefine the loss functions as a method of training the model. For example, its applicability extends to architectural, product, or automotive design. Training personalized models for generating designs may evolve into a requisite skill for designers.

The text-guided aesthetic diffusion model has some limitations in its functionality. For example, the model cannot directly specify the position of the generated objects in the design, and the generated design cannot exactly match the design site dimensions. Therefore, this method is most suitable for quickly establishing design concepts with clients, which designers can further refine and adjust to match the actual dimensions of the project site. This implies that the method needs to be used with manual intervention from designers to ensure the final design aligns with the requirements of the physical space. Despite these limitations, the method still holds significant potential and performs admirably in rapidly establishing design concepts with clients.

This study holds potential for further improvement, particularly regarding controllability. Enhanced controllability would facilitate generating results that more optimally align with design requirements. Thus, integrating a multilayer neural network into the diffusion model can enable precise control by constraining the generated outcomes. Hand-drawn sketches, color-block diagrams, and wireframe diagrams can all serve as modalities for governing image generation. Designers can fulfill the design requirements of diverse application scenarios by refining the diffusion model and amalgamating multiple control methods.

## Conclusions

Traditional interior design methods require designers to possess aesthetic awareness and professional knowledge while also dealing with tedious design tasks, leading to difficulties in achieving aesthetically pleasing designs and low design efficiency. To address these challenges, we proposed the aesthetic diffusion model. By allowing designers to input text descriptions, this model can generate a batch of visually pleasing interior designs, transforming the labor-intensive design process into a computer-generated one. To overcome the problem of limited training data, we first created an interior design dataset annotated with aesthetic labels. Then, a composite loss function was proposed that incorporates aesthetic scores, interior decoration styles, and spatial functions into consideration of the loss. Subsequently, the model was retrained using this dataset and the new loss function. Through this training, the model can generate aesthetically pleasing interior designs in batches based on text descriptions while also being able to specify the decoration style and spatial function of the design. Experimental results demonstrate that the proposed method in this research can, to a certain extent, replace the laborious creative design and drawing tasks required in traditional design, transforming the design process and significantly improving design efficiency.

This research also has some limitations. Firstly, for the generated interior designs, it is challenging to establish comprehensive quantitative evaluation metrics. Currently, we have referred to the literature and expert opinions to formulate some quantitative evaluation indicators, but further development of more evaluation dimensions is needed to achieve a more quantified assessment of subjective perceptions. Specifically, there is a need for the development of automated evaluation algorithms and benchmarks to achieve this goal. Secondly, our understanding of decoration styles may be limited by personal cultural influences, which may prevent us from fully objectively understanding the decoration styles of other countries. It would be beneficial to involve more designers from diverse cultural backgrounds in the data collection process to reduce cultural bias. Additionally, there is room for improvement in the level of detail in the generated images. More design details can be obtained by increasing the amount of training data and using higher image resolutions for training.

Future research can explore the following directions:

1. Hiring designers with diverse cultural backgrounds to establish a more comprehensive aesthetic interior design dataset to reduce the impact of cultural bias on the understanding of decoration styles.
2. In addition to relying solely on text guidance for image generation, it is possible to incorporate additional control mechanisms to achieve more precise control over the generated image results in the aesthetic diffusion model.
3. Researching the accuracy of dataset annotations. Currently, dataset annotations are often performed using either automated or manual methods, both of which have limitations. By combining these two annotation methods, the quality of the dataset can be improved, thereby enhancing the final generated design results.
4. Interior design evaluation relies heavily on manual assessments of aesthetics, decoration styles, and spatial functions. There is a pressing need to develop automated quantitative evaluation methods for assessing the generated interior designs.

## Data Availability

The datasets used and/or analysed during the current study available from the corresponding author on reasonable request.
